# An Identification Method for Upper Limb Spasticity Based on IDATC in Rehabilitation Training: A Preliminary Study

**DOI:** 10.3390/s26216964

**Published:** 2026-11-04

**Authors:** Zemiao Fang, Yungang Hao, Shuoyu Wang, Tao Liu

**Affiliations:** 1 State Key Laboratory of Fluid Power and Mechatronic Systems, School of Mechanical Engineering, Zhejiang University, Hangzhou 310027, China; 2 Zhejiang Key Laboratory of Additive Manufacturing Technology and Equipment, School of Mechanical Engineering, Zhejiang University, Hangzhou 310027, China; 3 Department of Intelligent Mechanical Systems Engineering, Kochi University of Technology, Kami-City 782-8502, Japan

**Keywords:** spasticity identification, stroke rehabilitation training, interaction force and movement monitoring, rehabilitation robot

## Abstract

**Highlights:**

**What are the main findings?**

- The negative peak of the index based on the divergence of acceleration and external torque changes (IDATC) is highly consistent with the EMG peak of the corresponding spastic muscle.
- The data used in this spasticity identification method based on IDATC are all current-state values, and an appropriate threshold value can enable this method to achieve a very high F1 score (preliminary result; the threshold was selected and evaluated using the same dataset, and needs further independent validation).

**What are the implications of the main findings?**

- It has potential to identify spasticity based on IDATC.
- This IDATC-based spasticity identification method has the potential to be used in rehabilitation devices or robots for real-time identification of spasticity onset during rehabilitation training.

**Abstract:**

During rehabilitation training, real-time monitoring and accurate identification of whether spasticity is occurring at the moment are crucial. This serves as the prerequisite for rehabilitation devices or robots to promptly implement interventions to alleviate spasticity, ensure patient safety, and ensure therapeutic efficacy. However, research on the real-time identification of the moment when the spasticity occurs by rehabilitation robots during training remains limited. In this paper, through clinical manifestations of spasticity and theoretical analysis, an index based on the divergence of acceleration and external torque changes (IDATC) and an IDATC-based method for identifying spasticity onset are proposed. A combined experiment was conducted to collect the kinematic, interaction force, and electromyography (EMG) data of stroke patients during spasticity provocation. The proposed spasticity identification method was validated using upper limb spasticity data collected from stroke patients. Preliminary results of the experiment demonstrate that the negative peak of the IDATC is highly consistent with the EMG peak of the corresponding spastic muscle, so it has potential to identify spasticity onset using the proposed IDATC-based spasticity identification method. Furthermore, provided that the threshold is appropriately selected, the proposed IDATC-based method may achieve an exceptionally high identification F1 score.

## 1. Introduction

Every year, more than 13.7 million people worldwide suffer from strokes [1,2]. Upper limb motor dysfunction is a common sequela of stroke. Up to 80% of stroke patients will lose some of their motor ability within six months after a stroke [3]. Due to the existence of neural plasticity, many patients can partially regain their lost motor ability [4,5]. Therefore, conducting sufficient rehabilitation training is an important treatment method for stroke survivors to restore their motor ability [6]. Spasticity is also a common sequela after stroke and is prone to occur during the rehabilitation process of stroke, with an incidence rate of 30–80% [7]. Clinically, it is characterized by hypertonia of the muscles, accompanied by exaggerated tendon twitching, which is caused by the excessive excitation of the stretching reflex after neurological diseases [8,9,10]. Clinically, post-stroke upper limb spasticity can be treated or alleviated through rehabilitation training such as stretching exercises, pharmacological treatment such as botulinum toxin injections, and physical therapy such as heat application, and surgical treatment [11,12,13]. In rehabilitation training, it is necessary to avoid triggering spasticity. During rehabilitation training, spasticity should be identified in time and treated immediately (e.g., reducing the speed of movement or stopping the training).

Currently, physiotherapists mainly subjectively identify the spasticity of the affected limb based on hand feel and assess the spasticity of the affected limb through scales [14,15,16,17]. This method is simple and easy to implement, requiring no additional equipment, but it is greatly influenced by subjective factors. Quantitative spasticity identification is mostly achieved through the analysis of surface electromyography (sEMG) [17,18,19]. This approach requires additional electromyography monitoring equipment and is complex to implement. Current research mainly focuses on the grading assessment of spasticity and benchmarking them against scales [20,21,22,23]. However there are relatively few studies on the identification of a sudden spasticity occurrence during rehabilitation training. In rehabilitation equipment or robots, the identification of a sudden spasticity occurrence during rehabilitation training is very important, as it serves as the prerequisite for rehabilitation devices or robots to promptly implement interventions to alleviate spasticity, ensure patient safety, and guarantee rehabilitation effects.

Therefore, we proposed an identification method for upper limb spasticity based on acceleration and interaction force variation in rehabilitation training by simultaneously monitoring the patient’s interaction force and upper limb movement. And an index based on the divergence of acceleration and external torque changes (IDATC) was proposed. When there is a deviation between the acceleration change and the interaction force change, it is considered that spasticity has occurred, avoiding misjudgment caused by the variable speed action of the rehabilitation training movement itself. For this, we conducted a preliminary study.

## 2. Materials and Methods

### 2.1. Spasticity Identification Methods

As the clinical manifestations of spasticity include increased muscle tone caused by excessive excitation of the stretching reflex after neurological diseases, which hinders traction movements, spasticity can be identified in real time by monitoring the conditions of muscle activities and limb movements.

**Figure 1:**
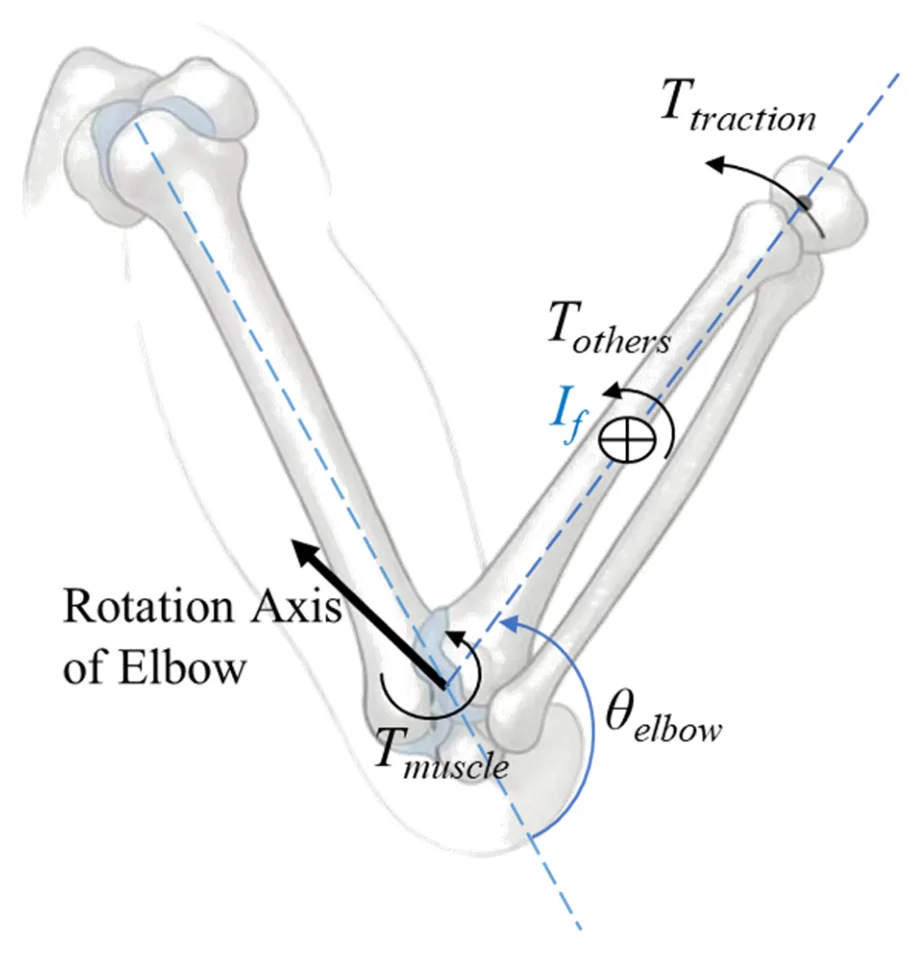
Schematic diagram of the torque acting on the elbow joint.

Taking the elbow joint as an example, as shown in Figure 1, the moment balance equation can be expressed as

(1) $$T_{\mathrm{traction}}+T_{\mathrm{muscle}}+T_{\mathrm{others}}=I_{f}{\ddot{\theta }}_{\mathrm{elbow}}$$

In the equation, *T_traction_* represents the torque exerted by the external traction at the elbow. *T_muscle_* represents the torque of muscle force at the elbow. *T_others_* represent the torque of other forces at the elbow, mainly affected by gravity. *I_f_* represents the moment of inertia of the forearm at the elbow. *θ_elbow_* represents the angle of the elbow joint, correspondingly, and ${\dot{\theta }}_{\mathrm{elbow}}$ and ${\ddot{\theta }}_{\mathrm{elbow}}$ are the angular velocity and angular acceleration of the elbow joint respectively. *θ_elbow_* can be calculated based on data obtained from IMUs or other means, and *T_traction_* can be calculated based on data from force sensors.

**Figure 2:**
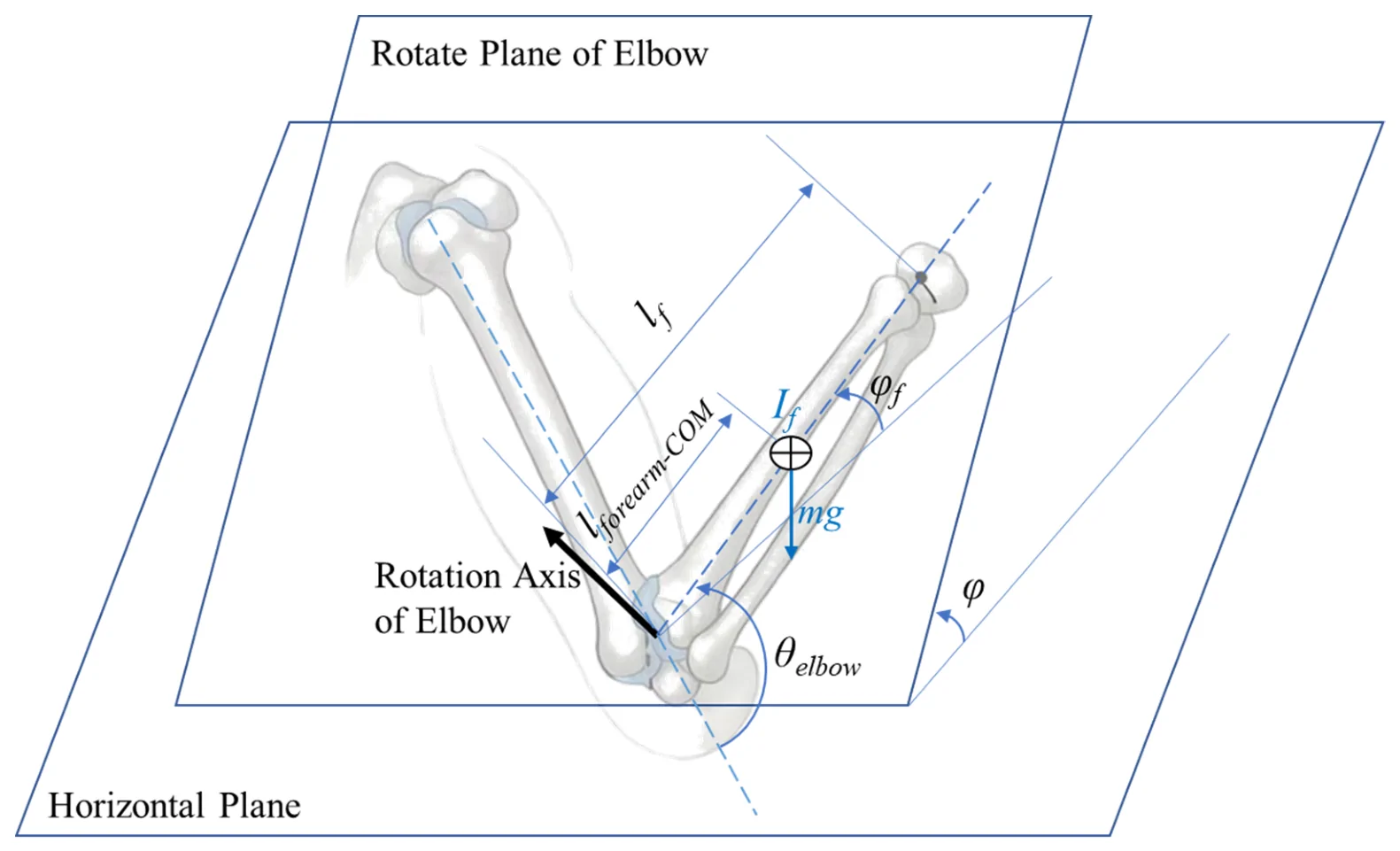
Schematic diagram of gravitational moment calculation.

As shown in Figure 2, the plane of forearm movement is the rotational plane of the elbow joint, perpendicular to the elbow joint rotation axis. The angle between this plane and the horizontal plane is denoted as *φ*. The angle between the forearm and the horizontal plane is denoted as *φ_f_*, the distance from the centroid of the forearm to the rotation axis of the elbow joint is denoted as *l_forearm_*_−*COM*_, and the mass of the forearm is denoted as m. Then, *T_others_* can be expressed as

*T_others_* = −*mgl_forearm_*_−*COM*_sin(*φ*)cos(*φ_f_*) (2)

In particular, when the plane of forearm movement is the horizontal plane (*φ* = 0) or the sagittal plane (*φ* = 90°), *T_others_* are simplified to 0 or −*mgl_forearm_*_−*COM*_cos(*φ_f_*) respectively.

The position of the center of mass (*l_forearm_*_−*COM*_), the moment of inertia (*I_f_*) and the mass (*m*) of the forearm used for calculating the torque were estimated through reference [24].

The external torque acting on the elbow joint is denoted as *T_external_*; then,

*T_external_* = *T_traction_* + *T_others_* (3)

Equation (1) can be simplified as

(4) $$T_{\mathrm{external}}+T_{\mathrm{muscle}}=I_{\mathrm{forearm}}{\ddot{\theta }}_{\mathrm{elbow}}$$

During the rehabilitation training process, the rehabilitation movements are known by the patients, who will make their best efforts to cooperate and complete these movements. According to Equation (4), the muscle activity can be indirectly estimated. By combining the relationship between movement and strength, the occurrence of spasticity can be estimated:

⬤ When the external torque *T_external_* is in the same direction as the velocity of the joint movement ${\dot{\theta }}_{\mathrm{elbow}}$. At this moment, the external torque and the patient’s rehabilitation movement are in the same direction, so it is in a traction motion.
  - If the direction of change in the external torque ${\dot{T}}_{\mathrm{external}}$ is the same as the direction of velocity ${\dot{\theta }}_{\mathrm{elbow}}$. At this moment, the external torque is increasing towards the direction of the patient’s rehabilitation movement; it is in a state of accelerating traction.
    · If the direction of the external torque change ${\dot{T}}_{\mathrm{external}}$ is the same as the direction of the joint movement acceleration change ${\dddot{\theta }}_{\mathrm{elbow}}$. At this time, both the external torque in the direction of the rehabilitation movement and the velocity of the rehabilitation movement increase simultaneously; it is considered that the muscles that hinder the rehabilitation movement are relaxed and no spasticity has occurred.
    · If the direction of change in the external torque ${\dot{T}}_{\mathrm{external}}$ is opposite to the direction of change in the acceleration of joint movement ${\dddot{\theta }}_{\mathrm{elbow}}$. At this point, the external torque in the direction of the patient’s rehabilitation movement is added, but instead, an acceleration that hinders the rehabilitation movement occurs. It is considered that spasticity has occurred.
  - If the direction of change in the external torque ${\dot{T}}_{\mathrm{external}}$ is opposite to the direction of velocity ${\dot{\theta }}_{\mathrm{elbow}}$. At this time, the external torque along the direction of the rehabilitation movement decreases, and the acceleration provided by the external torque for the rehabilitation movement diminishes.
    · If the direction of change in the external torque ${\dot{T}}_{\mathrm{external}}$ is the same as the direction of change in the acceleration of joint movement ${\dddot{\theta }}_{\mathrm{elbow}}$. At this point, the decrease in acceleration along the direction of the rehabilitation movement is related to the reduction in external torque in the same direction, rather than the patient obstructing the rehabilitation movement. No spasticity has occurred.
    · If the direction of change in the external torque ${\dot{T}}_{\mathrm{external}}$ is opposite to the direction of change in the acceleration of joint movement ${\dddot{\theta }}_{\mathrm{elbow}}$. At this moment, the external torque along the direction of the rehabilitation movement decreases, while the acceleration along the same direction increases, indicating that the patient is actively moving in the direction of the rehabilitation movement. It is considered that the movement is actively participated in and no spasticity has occurred.
⬤ When the external torque *T_external_* is opposite to the direction of the joint’s movement speed ${\dot{\theta }}_{\mathrm{elbow}}$. At this moment, the external torque directly opposes and decelerates the limb movement, and does not involve spasticity.

Based on this, we propose an index based on the divergence of acceleration and external torque changes (IDATC) to determine whether spasticity occurred in real time. The pseudo-code calculation process of this indicator is

Input: *T_external_*, ${\dot{T}}_{\mathrm{external}}$, ${\dot{\theta }}_{\mathrm{elbow}}$, ${\dddot{\theta }}_{\mathrm{elbow}}$,

*Begin:*

$\mathrm{IDATC}=\left\{\begin{array}{l} {\dot{T}}_{\mathrm{external}}{\dddot{\theta }}_{\mathrm{elbow}},T_{\mathrm{external}}{\dot{\theta }}_{\mathrm{elbow}}\geq 0\;\&\;{\dot{T}}_{\mathrm{external}}{\dot{\theta }}_{\mathrm{elbow}}\geq 0\\ \left|{\dot{T}}_{\mathrm{external}}{\dddot{\theta }}_{\mathrm{elbow}}\right|,\mathrm{others} \end{array}\right.$

*End.*

Output: *IDATC*.

To prevent 0 divided by 0 type distortion and reduce error sensitivity, symbol judgment is carried out by multiplication.

**Figure 3:**
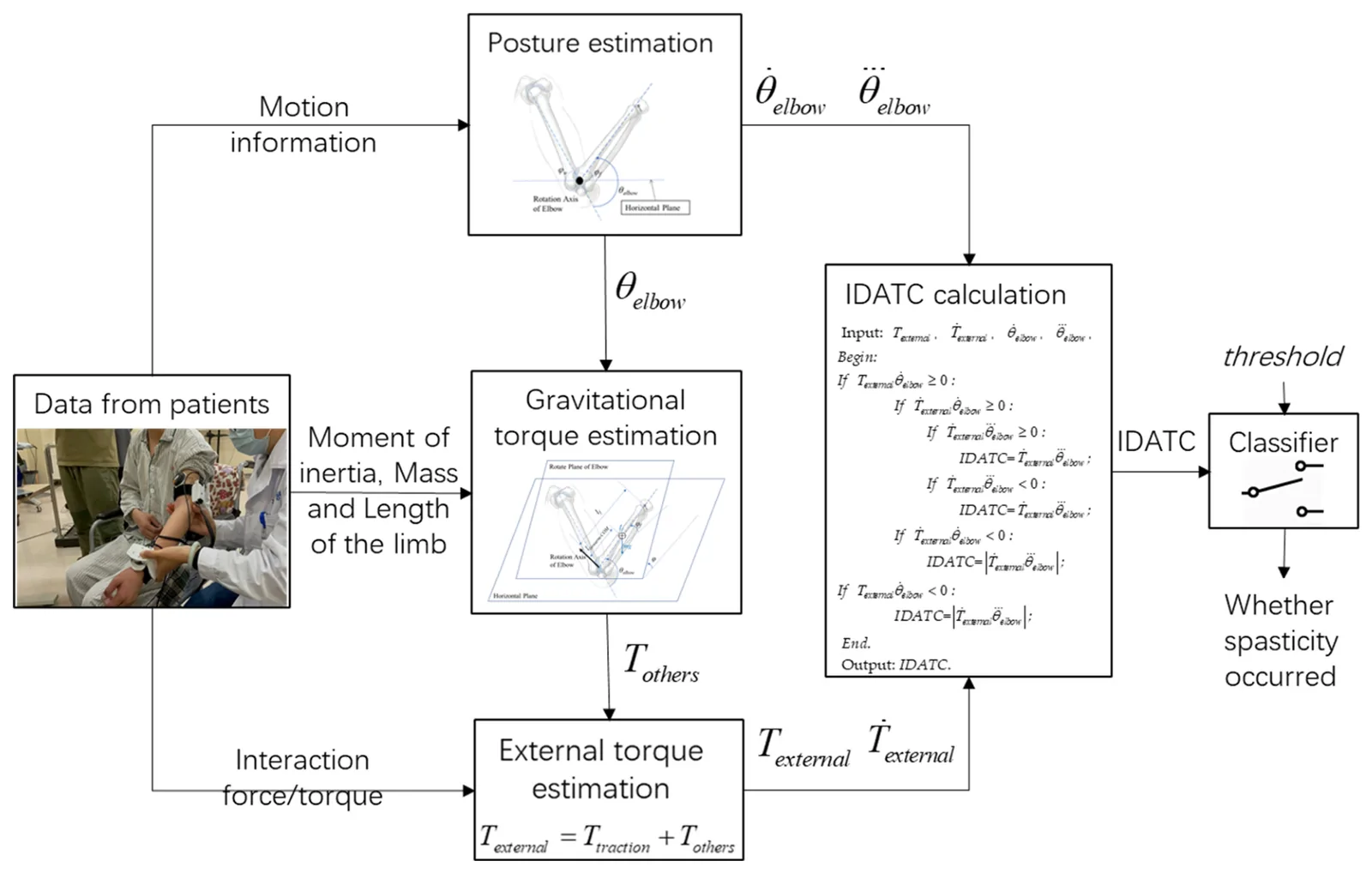
The structural diagram of the IDATC-based spasticity identification method.

Take a threshold: *threshold* > 0. If *IDATC* ≤ -*threshold*, it is considered that spasticity has occurred; otherwise, no spasticity has occurred. The structural diagram of the spasticity identification method is shown in Figure 3.

The data used in this identification method are all current-state values, and these parameters are readily accessible to rehabilitation devices or robots during the rehabilitation training process. Therefore, this method also has the potential to be used for rehabilitation devices or robots to identify spasticity onset in real time during rehabilitation training.

### 2.2. Experiments

We have jointly completed a clinical trial with researchers from UIUC to collect interaction force, electromyography and movement data of the affected limb when the spasticity was stimulated [25] (pp. 111–115). This study has been approved by the Medical Ethics Committee of Zhejiang Hospital with approval number AF/SC-06/04.2 and by the Medical Ethics Committee of the School of Biomedical Engineering and Instrument Science, Zhejiang University, under Application No. 2021-39. The experimental design is as follows.

#### 2.2.1. Experimental Setup

**Figure 4:**
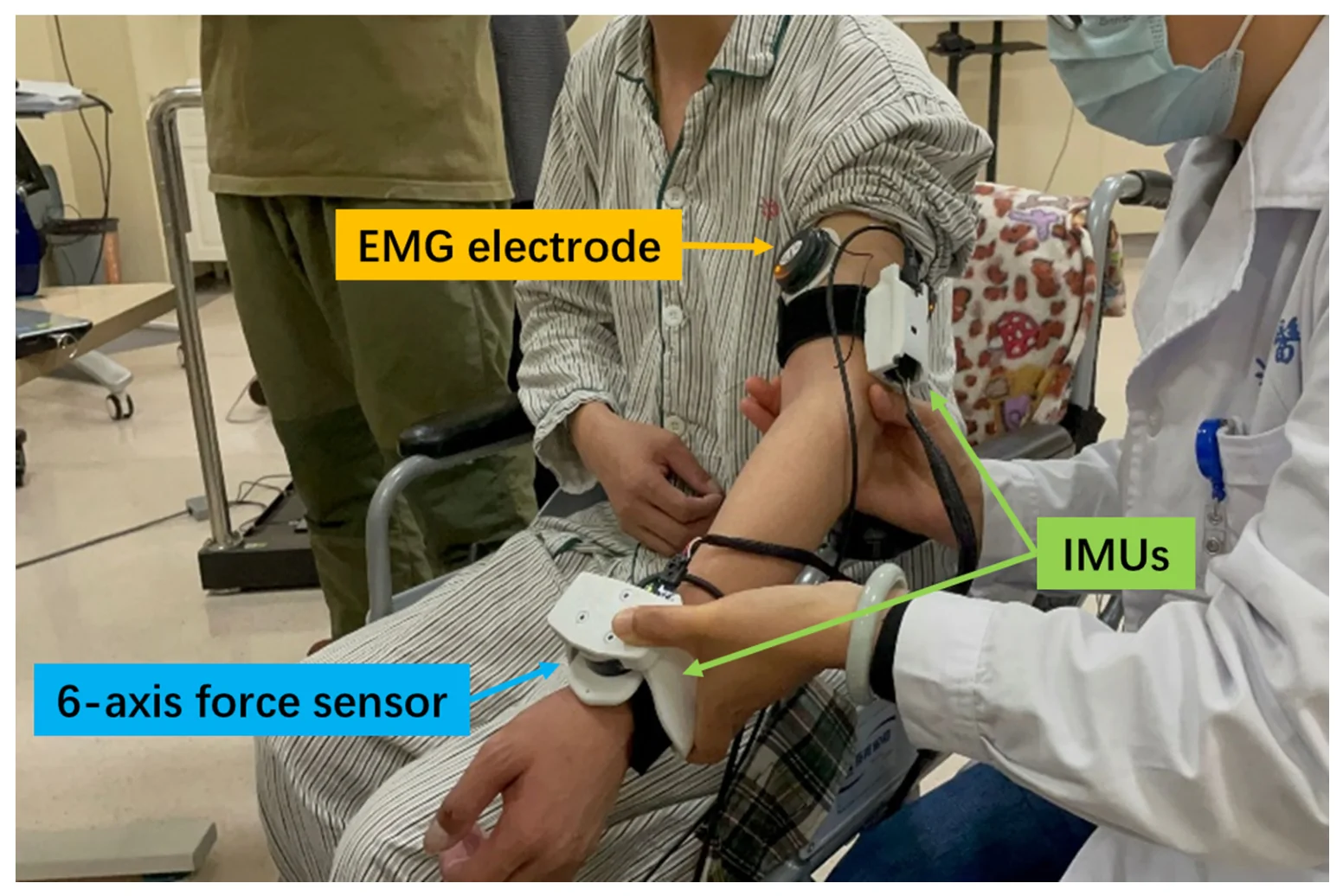
Sensor placements.

The team from UIUC designed a wearable multi-sensor experimental device, a Position, Velocity, and Resistance Meter (PVRM) [26,27], with the configuration shown in Figure 4. The device is portable and wearable, and the structural components connected to the sensor are made by 3D printing technology [28]. One IMU (MPU 6050, TDK InvenSense, San Jose, CA, USA, sampled at 100 Hz) was placed on the wrist and the other one on the upper arm to monitor the patient’s movement information. The traction module on the wrist is equipped with a 6-axis force sensor (M3703C, Sunrise Instruments, Nanning, China, sampled at 400 Hz) to monitor the interaction force between the physiotherapist and the patient during the traction operation. Electromyographic signals were collected through a wireless electromyographic system (Kine, Kiso Inc., Reykjavík, Iceland, sampled at 1600 Hz), and electromyographic signals are measured by attaching electromyographic sensor electrodes to the skin surface at the bicep and tricep positions of the subjects.

#### 2.2.2. Experimental Process

First, the maximum muscle strength of the patient’s upper limb is tested. The subject’s elbow is kept in a neutral position and one end is fixed with a tension ring. Then, the subject pulls the other end of the tension ring up or down with maximum force. The electromyogram (EMG) signals of the muscles that are exerting force at this time are recorded. The collected data can be used to normalize the subsequent data measured by patients with different constitutions.

Then, the patient is fitted with electromyographic signal sensors, IMUs and a multi-dimensional force sensor. Under the guidance of a rehabilitation therapist, the affected elbow joint of the subject was slowly (i.e., velocity <30°/s) moved back and forth five times. Then, the biceps brachii and triceps brachii were rapidly stretched (using the therapist’s preferred fast velocity) three times each. The biceps brachii and triceps brachii are a pair of antagonistic muscles. During rapid stretching, after completing the rapid stretching and reaching the maximum stretching position, the antagonistic muscles are slowly stretched to bring the elbow joint back to the initial rapid stretching position. When spasticity occurred, stretching is slowed until the maximum stretching position is reached to relieve the spasticity.

During the rapid stretching process, the spasticity of the affected limb may be triggered. The kinematic and interaction force information during the experiment is recorded for spasticity identification by the proposed method. Surface electromyography signals are used to determine whether spasticity really occurred, serving as a reference to verify the accuracy of the proposed method in spasticity identification. Each stretching process is regarded as an event, and on this basis, the identification result of the proposed method is counted.

After the subject completes their experimental movements, the IMU and 6-axis force sensor assembly, as well as the electromyography sensor, are removed from the subject. The electromyography electrodes are placed on a hard desk surface, and the assembly of the IMU and 6-axis sensor was used to gently tap the electrode three times in succession. Subsequently, data collection from the IMU, 6-axis force sensor, and electromyography sensor was terminated. During the final gentle tapping process, three abnormal peaks were generated simultaneously in the data from different types of sensors. In the data processing stage, these three abnormal peaks were found and aligned to achieve data synchronization.

Eligible subjects were required to be aged between 18 and 80 years, have impaired upper limb motor function due to stroke, and have signed the informed consent form by themselves or their legal guardians.

## 3. Results

### 3.1. Data Processing

**Figure 5:**
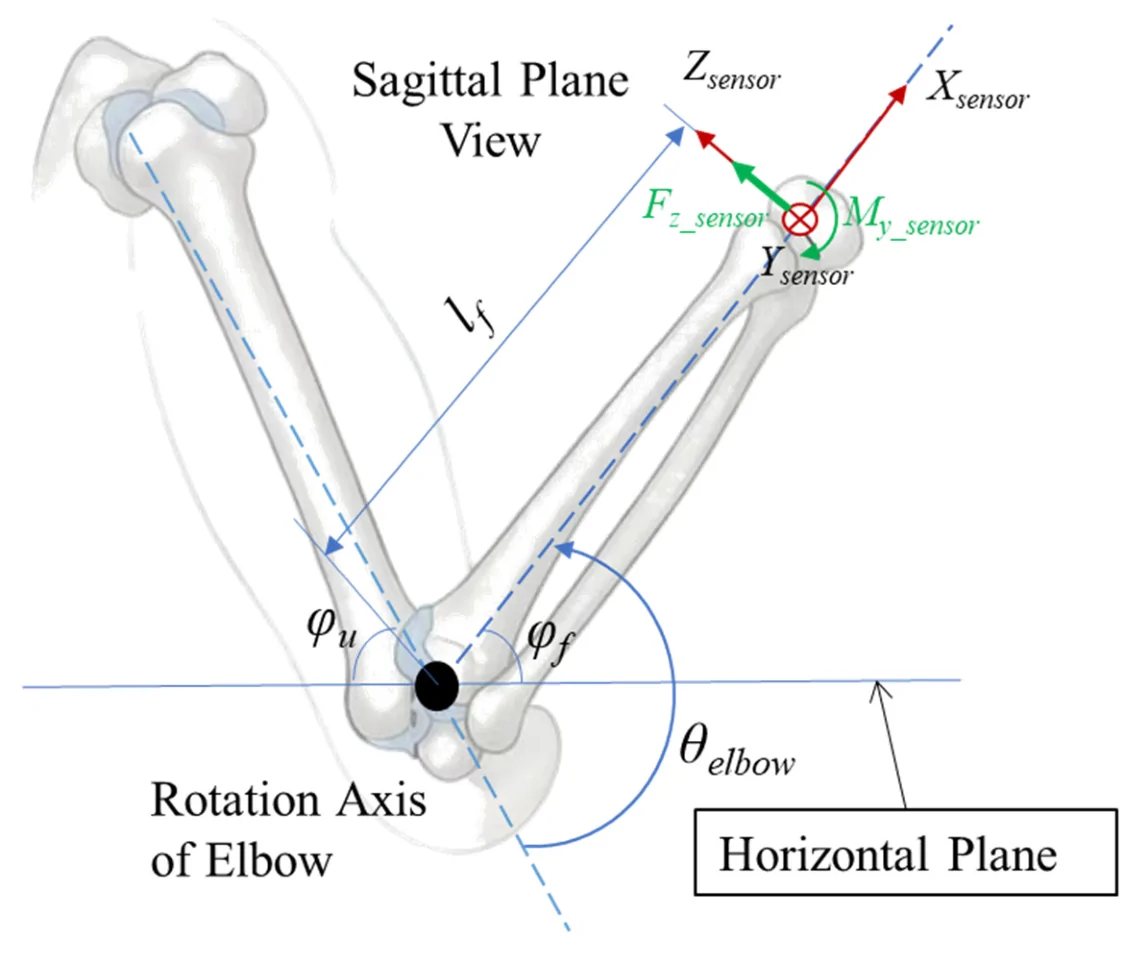
Schematic diagram of elbow joint movement in the sagittal plane.

The movement of the elbow joint in the experiment was completed in the sagittal plane of the patient, as shown in Figure 5.

The angle between the forearm and the horizontal plane is denoted as *φ_f_*, and the angle between the upper arm and the horizontal plane is denoted as *φ_u_*. *φ_f_*, *φ_u_* and their angular velocity can be obtained from the IMU used in the experiment. Since the movement in the experiment was located in the sagittal plane of the subject, obviously, the elbow joint angle *θ_elbow_* is

*θ_elbow_* = *φ_f_* + *φ_u_* (5)

The external torque *T_external_* is

*T_external_* = *T_traction_* + *T_others_* = *T_traction_* − *mgl_forearm_*_−*COM*_cos(*φ_f_*) (6)

In this experiment, the position of the 6-axis force sensor and its coordinate system are shown in Figure 5, and the traction torque in Equation (6) is

*T_traction_* = *F_z_*__*sensor*_*l_f_* − *M_y_*__*sensor*_ (7)

All data was filtered by a fourth-order Butterworth filter with cut-off frequency of 4 Hz for angular position, velocity, and acceleration signals, and 15 Hz for force and torque signals.

Change in the external torque ${\dot{T}}_{\mathrm{external}}$ and change in the joint acceleration (angular jerk) ${\dddot{\theta }}_{\mathrm{elbow}}$ are obtained by first-order and second-order numerical differentiation of the external torque and the angular velocity respectively.

The filtering and numerical differentiation processes only utilize current and historical data.

EMG signals were detrended, notch-filtered (at 50 Hz), bandpass-filtered (fourth-order Butterworth filter at 10–500 Hz), rectified, and were normalized using the EMG peak values obtained in the first step of Section 2.2.2. During passive stretching, spastic muscles experienced explosive increases in EMG due to exaggerated stretch reflexes, while active movement does not promote this. If the resulting signals exceed 20% for less than 0.5 s, it is considered that spasticity has occurred during the corresponding stretch. If the resulting signals exceed 20% for more than 0.5 s, it is considered that the corresponding stretch contains voluntary muscle contraction and does not involve spasticity. And other EMG conditions (not exceeding 20% of the peak level in the first step of Section 2.2.2) are considered as not experiencing spasticity. The process of spasticity identification using EMG signals only relies on EMG signals, and the results are used as a reference for determining whether spasticity really occurs.

The true positive (TP), false positive (FP) and false negative (FN) of the proposed method in spasticity identification are defined:

- True positive (TP): Spasticity is identified as spasticity.
- False positive (FP): Non-spasticity is identified as spasticity.
- False negative (FN): Spasticity is identified as non-spasticity.

To describe the performance of the proposed method in spasticity identification, the precision rate, detection rate (recall) and F1 score in statistics are introduced:

(8) $$\mathrm{Precision}=\frac{\mathrm{TP}}{\mathrm{TP}+\mathrm{FP}}$$

(9) $$\mathrm{Recall}=\frac{\mathrm{TP}}{\mathrm{TP}+\mathrm{FN}}$$

(10) $$F1=\frac{2\times \mathrm{Precision}\times \mathrm{Recall}}{\mathrm{Precision}+\mathrm{Recall}}$$

The precision rate can describe whether all the events identified as spasticity are true spasticity events to prevent misidentification. The detection rate (recall) can describe whether all spasticity events are identified as spasticity events to prevent missed identification. Both parameters are crucial to the performance of the proposed method; hence, the F1 score is employed for comprehensive description.

### 3.2. Preliminary Results

Eight subjects participated in this experiment, and their statistical information is presented in Table 1.

**Figure 6:**
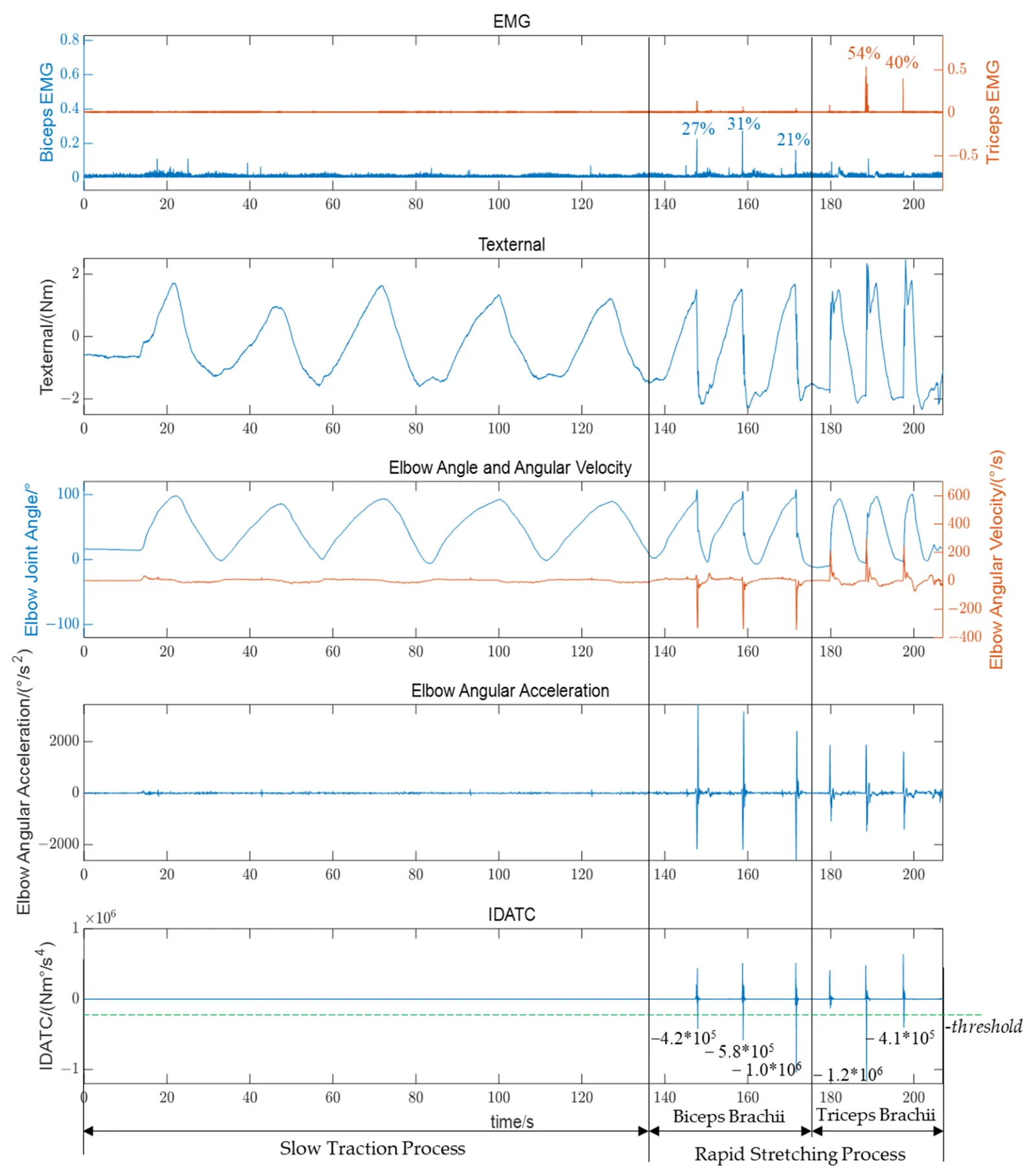
The electromyography, external torque, movement data of the affected limb and the index IDATC we proposed of a patient during the experiment. The green dashed line represents *-threshold.*

As shown in Figure 6, the electromyography, external torque, movement data of the affected limb and the index *IDATC* we proposed of a patient (Subject 5) during the experiment are presented for demonstration. In the EMG subgraph, the blue left axis represents the EMG of the biceps brachii, and the orange right axis represents the electromyography of the triceps brachii. The electromyography signals have been normalized. In the subgraph of elbow angle and angular velocity, the blue left axis represents the elbow joint angle, and the orange right axis represents the angular velocity of the elbow joint. For clarity, the external torque, the angular acceleration of the elbow joint, and the index *IDATC* we proposed are drawn separately in three subplots. The timelines of all subgraphs have been aligned.

During the slow traction process, no spasticity occurred in the affected limb, and the changes in EMG and kinematic data were gentle. The proposed index based on the divergence of acceleration and external torque changes did not show any abnormalities. During the rapid stretching process, the affected limb experienced spasticity, sharp peaks appeared in the EMG, and both the movement speed and acceleration of the affected limb underwent drastic changes. The proposed index *IDATC* significantly showed a negative peak when spasticity occured.

The negative peak of the IDATC is highly consistent with the EMG peak of the corresponding spastic muscle. In this experiment, in terms of real-time identification of spasticity occurrence, its features are evidently more distinct than those of angular velocity, angular acceleration, or external torque alone. For example, in the trial of Figure 6 at the rapid stretching process near 180s, when only considering angular velocity, angular acceleration, or external torque, their behaviors are similar to those observed during rapid stretching with spasticity. However, the EMG reference indicates no spasticity, and the result of the proposed method is consistent with the EMG reference results. This also indicates that the significant negative peaks in IDATC are not induced solely by rapid stretching or by data processing.

In the experiment, the biceps and triceps brachii were stretched (5 + 3 × 2) × 8 = 88 times (slow 5 × 8 + 3 × 8 = 64 times, rapid 3 × 8 = 24 times) separately (88 × 2 = 176 in total). A total of (5 + 3) × 2 × 8 = 128 slow stretches were performed (64 times each for the biceps and triceps), and no spasticity was caused. In total, rapid stretching was performed 3 × 2 × 8 = 48 times (24 times each for the biceps and triceps) to trigger spasticity, causing 39 spasticity events (20 times for the biceps and 19 times for the triceps). So the total number of spasticity events is 39. The reference spasticity events were determined by EMG signals.

Here, the threshold we set is 2.0 × 10^5^ (-*threshold* = −2.0 × 10^5^) Nm°/s^4^. Under this threshold, the F1 score of spasticity identification reaches 100%, indicating that the proposed method is highly feasible for rehabilitation equipment to identify spasticity in real time during the rehabilitation process (preliminary result; the threshold was selected and evaluated using the same dataset, and needs further independent validation). The issue of threshold selection will be discussed in the next section.

## 4. Discussion

### 4.1. The Rationality of Identifying Spasticity Based on IDATC

**Figure 7:**
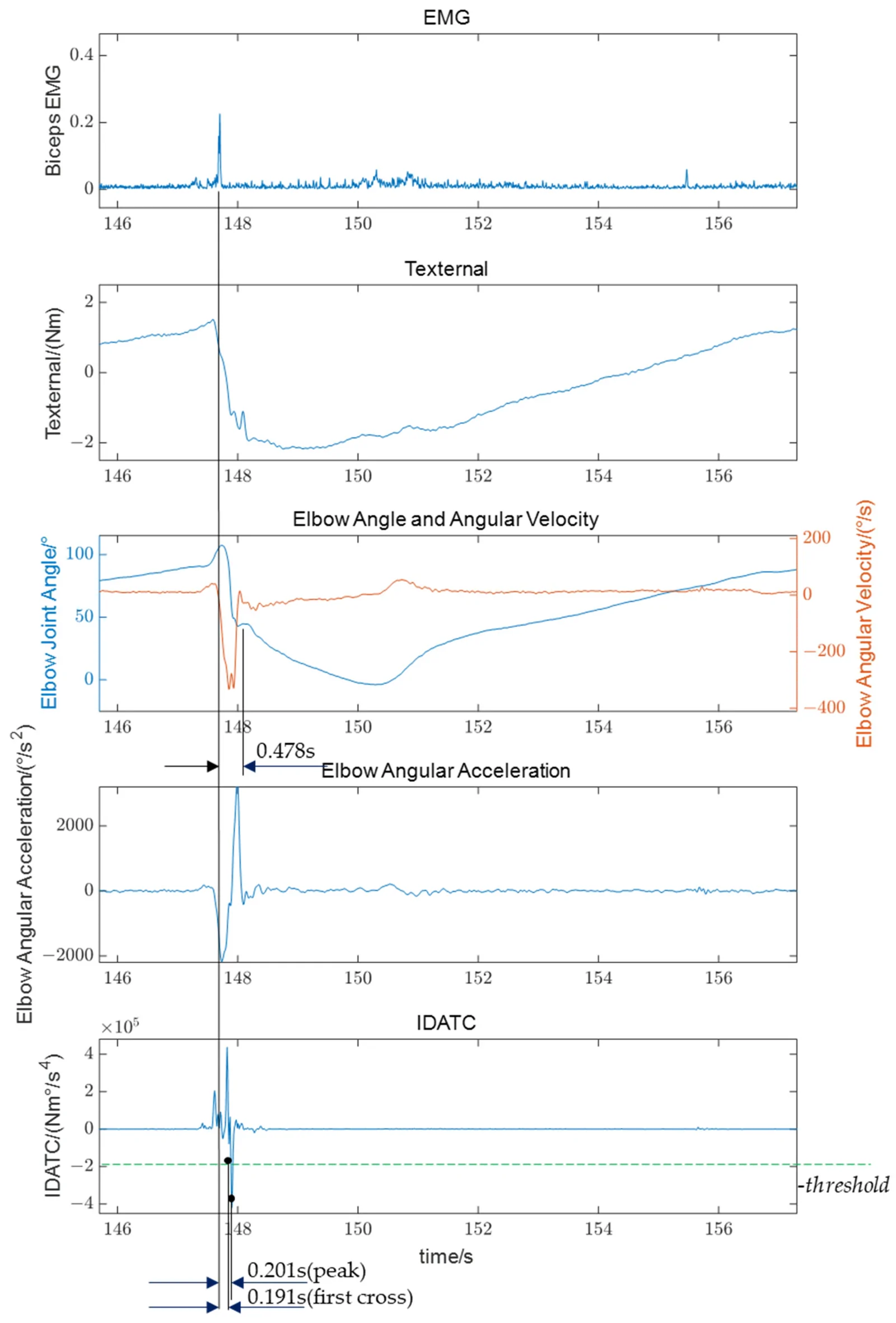
The electromyography, external torque, movement data of the affected limb and the index IDATC we proposed in one of the biceps brachii spasticity events (the first spasticity event in Figure 6). The green dashed line represents *-threshold.*

As shown in Figure 7, it is a process of rapidly stretching the biceps brachii and triggering spasticity. After rapidly stretching the biceps brachii, an angular acceleration in the direction of the elbow joint angle reduction is rapidly generated, and the angular velocity surges in the direction of the elbow joint angle reduction. During this process, the biceps brachii spasms and peaks in the electromyographic signals appear. Subsequently, the muscle begins to spasm and affects the movement of the affected limb. Then the physiotherapist senses the change in hand feel, and applies a constant-speed slow stretching treatment to relieve the spasticity immediately.

In this spasticity, the delay from the peak of the spasticity EMG signal to the appearance of the negative peak of the IDATC was 0.201 s. Based on the statistics of 39 identified spasticity events, the mean value of this value in spasticity during the experiment was 0.165 s, and the standard deviation was 0.044 s. Taking the influence of Electromechanical Delay (EMD, the time from the appearance of EMG signals to the generation of muscle movements) [29,30,31] into account, the time when the negative peak of the IDATC appears is consistent with the time when the mechanical movement of the spasticity occurs. The negative peak of the IDATC points to the moment when the spasticity occurs. It is reasonable to identify spasticity based on IDATC.

For spasticity identification based on IDATC, the spasticity event is judged when IDATC first crosses the threshold, which occurs earlier than the peak of IDATC. In this experiment, using a threshold of 2.0 × 10^5^ Nm °/s^4^, the mean delay from the electromyographic peak to IDATC first crossing the threshold was 0.151 s, with a standard deviation of 0.042 s. In line with clinical practice during rehabilitation training, where spasticity is managed immediately upon detection, the proposed method identifies a spasticity episode within a single stretching event (i.e., a single movement) as soon as IDATC is first detected crossing the threshold.

However, it should be noted that although the proposed method only uses current and historical data, this delay data does not include the time consumed by real-time data processing.

The delay from the peak of the EMG signal of the spasticity to the treatment by the physiotherapist in this spasticity was 0.478 s. Only when the total response time of the real-time rehabilitation system (the sum of IDATC delay, IDATC computation time, and rehabilitation device response time) is comparable to this can a therapist-level spasticity response be attained.

### 4.2. Threshold Selection Problem for Identifying Spasticity Based on IDATC

In this experiment, the maximum IDATC of its negative peaks in the 39 spasticity events was −4.0 × 10^5^ Nm°/s^4^, and the minimum IDATC was −1.3 × 10^5^ Nm°/s^4^ at other times. Therefore, taking a threshold that satisfies 1.3 × 10^5^ < *threshold* < 4.0 × 10^5^ (Nm°/s^4^) can produce a 100% F1 score in identification spasticity occurrence moments. In this experiment, if the threshold is less than 1.3 × 10^5^ (-*threshold* > −1.3 × 10^5^) Nm°/s^4^, non-spastic periods may be mistakenly identified as spasticity occurrences, and the precision rate will decrease. If the threshold is greater than 4.0 × 10^5^ (-*threshold* < −4.0 × 10^5^) Nm°/s^4^, some spasticity moments will not be identified, and the detection rate (recall) will decrease.

The adjustment of the threshold determines the sensitivity of the identification results to spasticity. The lower the threshold, the more sensitive it is. However, if the threshold is set too low, some moments when spasticity does not occur may be mistakenly identified as spasticity (affects the precision rate). Conversely, the higher the threshold, the more sluggish it becomes. If the threshold is too high, some moments of spasticity may not be identified (affects the detection rate).

In the future, with more clinical data on patient spasticity, the selection range of the threshold can be further optimized. Furthermore, if the analyzed sample data are sufficiently abundant, different thresholds might be selected based on the individual conditions (such as spasticity grade, level of tension, muscle strength, etc.) of each patient, rather than applying a fixed threshold.

### 4.3. Limitations

#### 4.3.1. The Same Dataset Was Used for Threshold Selection and Performance Evaluation

The key limitation of this study is that the threshold selection and performance evaluation of the proposed method were performed on the same dataset. This may lead to overfitting and result in overly optimistic performance estimates. The threshold was essentially tuned on the entire dataset, so the results cannot reliably reflect the method’s generalization capability to independent, unseen data. Future studies should adopt an independent external dataset to validate the proposed method.

#### 4.3.2. Applicability of the Proposed Method in Low-Angular-Velocity Conditions Has Not Been Verified

The performance of the proposed method under low-speed conditions has not been validated. In fact, spasticity is less likely to occur under low-angular-velocity conditions. In the experiment, we also did not observe spasticity during the slow stretching processes (velocity < 30°/s). Therefore, this may be a limitation of the proposed method.

#### 4.3.3. Real-Time Performance

As demonstrated in Section 4.1, the delay from the EMG peak to either the IDATC peak or the first threshold crossing is comparable to the typical EMD, which implies that the proposed IDATC aligns well with the real-time onset of spastic mechanical features. However, it should be noted that this delay does not incorporate the time consumption of the identification process itself and therefore cannot represent the time delay from the onset of spastic mechanical features to the spasticity being actually identified. In addition, the exposition in Section 2.1 indicates that the proposed method uses only current and past data for spasticity identification. These two points suggest that the proposed method has the potential for real-time identification, but the intrinsic time expenditure of the method itself has not been validated.

In practical applications, differences in kinematic or kinetic data calculations arising from the use of different types of sensors or varying sensor placement, variations in computational complexity of the gravitational torque component of the external torque across different body postures, and differences in the computing power of the application platform will all affect the time required for the proposed method to identify spasticity. Therefore, the real-time performance of the proposed method still requires further verification.

#### 4.3.4. Other Limitations

The proposed method is analyzed and validated using the elbow joint as an example. Although it can be theoretically extended to other upper limb joints, due to the complexity of the human body, it is necessary to verify the applicability of the proposed method on other joints in the upper limbs. Similarly, the performance of the proposed method across different rehabilitation training movements and tasks also requires verification.

Furthermore, due to the limited size of the clinical sample, the validation of the proposed method may be insufficient. Factors such as different subject populations, varying clinical disease courses, and different sensor types and placements may all affect the method’s performance metrics, such as delay and F1 score (precision rate, detection rate).

## 5. Conclusions

Based on the clinical manifestations of spasticity and theoretical analysis, we proposed the IDATC and a method of real-time identification for upper limb spasticity based on acceleration and interaction force variation in rehabilitation training (IDATC-based). Validated by upper limb spasticity data collected from eight stroke patients, the proposed method proved to have the potential for spasticity identification. Furthermore, provided that an appropriate threshold is selected, it may achieve exceptionally high identification F1 score (preliminary result; the threshold was selected and evaluated using the same dataset, and needs further independent validation). However, the proposed method still has limitations. So before the proposed method wider application in rehabilitation robots, further validation is necessary in a larger, more heterogeneous stroke population and across different rehabilitation tasks.

## Figures and Tables

**Table 1:** Subject demographic information.

Subject ID	Age/Year	Gender	Affected Side	Biceps MAS	Triceps MAS	Post Injury
1	49	Male	Right	0	1	2 months
2	36	Male	Left	2	2	3 months
3	79	Female	Left	1	1	3 months
4	61	Male	Left	2	2	6 years
5	60	Male	Right	2	2	2 years
6	68	Male	Left	2	0	1 month
7	57	Female	Left	2	3	7 years
8	72	Female	Left	1	3	9 years

## Data Availability

Data are unavailable due to privacy restrictions.

## Abbreviations

The following abbreviations are used in this manuscript:

IMU	Inertial Measurement Unit
EMG	Electromyogram
PVRM	Position, Velocity, and Resistance Meter
IDATC	Divergence of Acceleration and External Torque Changes
EMD	Electromechanical Delay
